# Estimation of apparent thermal inertia of roofing materials from aerial thermal imagery

**DOI:** 10.1038/s41598-024-64371-3

**Published:** 2024-07-10

**Authors:** Emanuele Mandanici, Gabriele Lo Grasso, Maria A. Tini, Antonio Zanutta

**Affiliations:** Department of Civil, Chemical, Environmental and Materials Engineering (DICAM), University of Bologna, viale del Risorgimento 2, 40136 Bologna, Italy

**Keywords:** Civil engineering, Imaging and sensing

## Abstract

The rapid expansion of urban areas and soil sealing is enhancing the Urban Heat Island (UHI) phenomenon, especially during heat waves. The different thermal inertia of the building materials compared to natural surfaces is one of the major driving factors of UHI. The present contribution aims to test a methodology for mapping the Apparent Thermal Inertia (ATI)—a proxy that can be derived from remote sensing data—of roofing surfaces at the scale of an entire city and with a high spatial resolution. Day and night aerial thermal images with the resolution of 0.5 m were acquired over two test areas in Bologna (Italy), together with satellite multispectral data. Statistics on the buildings in the test areas are computed considering different classes of roofing materials (e.g. bituminous sheath, clay tiles, metal sheet, gravel tiles). Observed median ATI values for each class range from 0.03 to 0.09 K$^{-1}$ with interquartile ranges between 0.02 and 0.14 K$^{-1}$, so the intra-class variability in some cases appears higher than the variability among different material classes, proving the importance of ATI mapping for UHI investigations.

## Introduction

Global urbanization phenomena have been generating a rapid expansion of urban areas^1^, which in turn have brought out a significant environmental and social phenomenon known as the Urban Heat Island (UHI) effect. UHI refers to the higher temperature in urban areas compared to their surrounding rural regions^2,3^. Many factors contribute to the UHI effect, the reduction of green areas and consequently of the evapotranspiration rate, the higher heat capacity of building materials compared to natural surfaces, the geometry of urban areas creating canyons and multiple reflections of longwave radiations, the anthropogenic heat produced by air conditioning systems, gaseous emissions and atmospheric pollution^4^. UHI has significant impacts on the energy demand of our cities and on people’s living standards, especially considering the increasing frequency and intensity of heat waves as a consequence of climate change^5,6^. For this reason, UHI drivers must be investigated and taken into account for a careful urban planning, in the perspective of the development of “cool cities”.

Building materials play an important role in determining the urban microclimate^7^, because they can increase significantly heat retention, according to the different capacity of absorbing and emitting heat. Among the properties which describes the thermal behaviour of the materials, thermal inertia is particularly meaningful for UHI prediction and mitigation. For example, a study on the cities of Bhopal and Guwahati in India found a clear correlation between the difference in thermal inertia for urban and rural areas and the magnitude of the surface temperature observed with MODIS satellite data^8^. In particular, the difference in thermal inertia shows a positive correlation with surface UHI during nighttime as stored heat is released during the night^9^. During daytime the situation is opposite, because the temperature of the urban surfaces rises slower compared to rural surfaces, as a consequence of higher thermal capacity^9^. Such studies analysed these effects at a large scale, due to the coarse resolution (1 km) of the used satellite imagery. However, a finer resolution is desirable to study the microclimatic variability inside urban centres.

Theoretically, Thermal Inertia (TI) is defined as,

1 $$\begin{aligned} TI = \sqrt{C \cdot \rho \cdot k} \end{aligned}$$

where *k* is the thermal conductivity, $\rho$ is the density and *C* is the heat capacity^10^. Unfortunately, these parameters are very difficult to be measured extensively at the scale of an entire city, because they require dedicated in situ measurements. Models for direct computation of thermal inertia from multispectral thermal data were developed for the THEMIS instrument, onboard the Mars Odyssey spacecraft, for the exploration of Mars^11^. However, these models are not suitable for Earth observation applications in urban areas, because they are tuned for Martian soils and atmosphere and have a 20% overall accuracy^11^.

To overcome the limitation of an exact computation of the thermal inertia, another quantity has been proposed in the literature which can serve as an indicator: the Apparent Thermal Inertia (ATI). Traditionally, it is defined as follows^10^,

2 $$\begin{aligned} ATI = S \dfrac{1 - A}{\Delta T} \end{aligned}$$

where $\Delta T$ is the diurnal temperature amplitude computed from the difference between maximum and minimum surface temperature observations, *S* is a correction factor which accommodates for different solar illumination conditions (e.g. seasonal variations), and *A* is the overall albedo of the surface.

The solar correction factor *S* can be estimated referring to Van doninck et al.^12^ as follows,

3 $$\begin{aligned} S = sen{\phi } sen{\delta } \cdot (1- tan^2\phi tan^2\delta )^{\tfrac{1}{2}} + cos{\phi } cos{\delta } \arccos ({-tan{\phi } tan{\delta }}) \end{aligned}$$

where $\phi$ is the latitude and $\delta$ is the solar declination.

Actually, the albedo is another important factor affecting urban microclimate. It is defined as the ratio between the surface upwelling flux and the downward solar flux (direct and diffuse)^13^. Generally speaking, it is a critical parameter for Earth’s climate due to its impact in the radiation budget, and recently its implications for the urban microclimate have been investigated^14^. In particular, the total shortwave broadband albedo, i.e. the spectral albedo integrated over the whole shortwave range, is often considered. According to a widely adopted simplified approach^13,15,16^ it can be computed as a weighted average of narrowband spectral reflectance values from a multispectral sensor, assuming the downward flux distribution as a weighting function and a Lambertian behaviour:

4 $$\begin{aligned} A = \sum w_\lambda \rho _\lambda \end{aligned}$$

where $\rho$ is the spectral reflectance from a band and *w* is the weight. The albedo enters also in the computation of ATI (as for Eq. 2) to compensate for the different absorption of solar radiation^10^.

ATI can be computed from remote sensing data, provided that at least two surface temperatures are measured to quantify the diurnal temperature fluctuations.

Despite its early definition in the Seventies^17^, ATI has been widely used only in remote sensing applications for precision agriculture and soil moisture mapping^18,19^ and for planetary exploration^20,21^. ATI was used for the evaluation of soil moisture content for hydrological purposes using a temporal series of MODIS satellite imagery (1 km resolution)^22^. The idea behind the proposed method is that, when water content increases in soils, ATI also proportionally increases, resulting in a reduction of diurnal temperature fluctuation of the soils^22^. ATI derived on a pixel basis from MODIS images was used also for geological mapping, with the intent of delineating the major rock types, on the assumption that a proxy of thermal inertia is influenced by density^23^. Recently, ATI was used as a proxy of thermal inertia to investigate the surface of Mars^24^. The evaluation of the temperature difference was calculated starting from thermal imagery acquired by THEMIS, one during the day time and another during night time^24^.

Instead, there is a very limited usage of this parameter in urban environments, mostly with coarse resolution satellite data (1 km)^9^, therefore the knowledge of ATI values for building materials is still poor. Mangiameli et al.^25^ used satellite images from Sentinel-2 and MODIS to study UHI in the city of Catania (Italy), evaluating in particular the difference from day-time to night-time in land and air temperatures, also in terms of different spatial resolution; however, their study does not involve ATI. Gaitani et al.^26^ analyzed the thermal characteristics of materials and temperature distribution of objects at ground using multispectral imaging sensors mounted on aerial platforms and in-situ measurements for the municipality of Ymittos (Greece)^26^. However, ATI values for specific materials are not reported.

This lack of information at urban scale is obviously related to the spatial resolution of satellite thermal imagery, which is often inadequate for the complexity of urban structure, and to the logistics and costs of performing aerial surveys. However, the development of new platforms and the advances of thermal imaging technology may change the paradigm in the near future. For example, Albedo Space Corporation announced its plans for the new Albedo mission, which will include a new thermal sensor with a nominal ground sampling distance of 2 m^27^, an unprecedented spatial resolution allowing new applications for the urban environment.

While in rural areas ATI is generally used to infer other parameters of interest from remote sensing, such as soil moisture content^28^, in urban areas it can be a key indicator in determining the energy performance of roofing materials and may help the decision makers in preferring specific materials for construction or maintenance purposes.

Considering the current lack of information about the spatial variation of ATI in urban areas, in particular variations among different roofing materials and variability within the same kind of material, a method to map ATI at the city scale is to be developed. Indeed, the present paper proposes a methodology to compute ATI over building roofs surfaces and to infer statistics for some of the most common roofing materials of the city of Bologna. The methodology exploits the acquisition of thermal images at high spatial resolution from two aerial surveys, one performed during the day and the second in the night. It combines photogrammetric techniques, which are necessary to generate mosaics of thermal images with accurate geolocation and metric proportions, and temperature calibration procedures, which are used to estimate accurate temperature differences between day and night. Finally, the evaluation of the key parameters for building roofs, such as albedo, sky-view factor and ATI, is carried out in GIS environment. Overall, the proposed method is designed to maximize the information achievable from remote sensing data whilst limiting the need of ground surveys.

## Materials and study area

**Figure 1:**
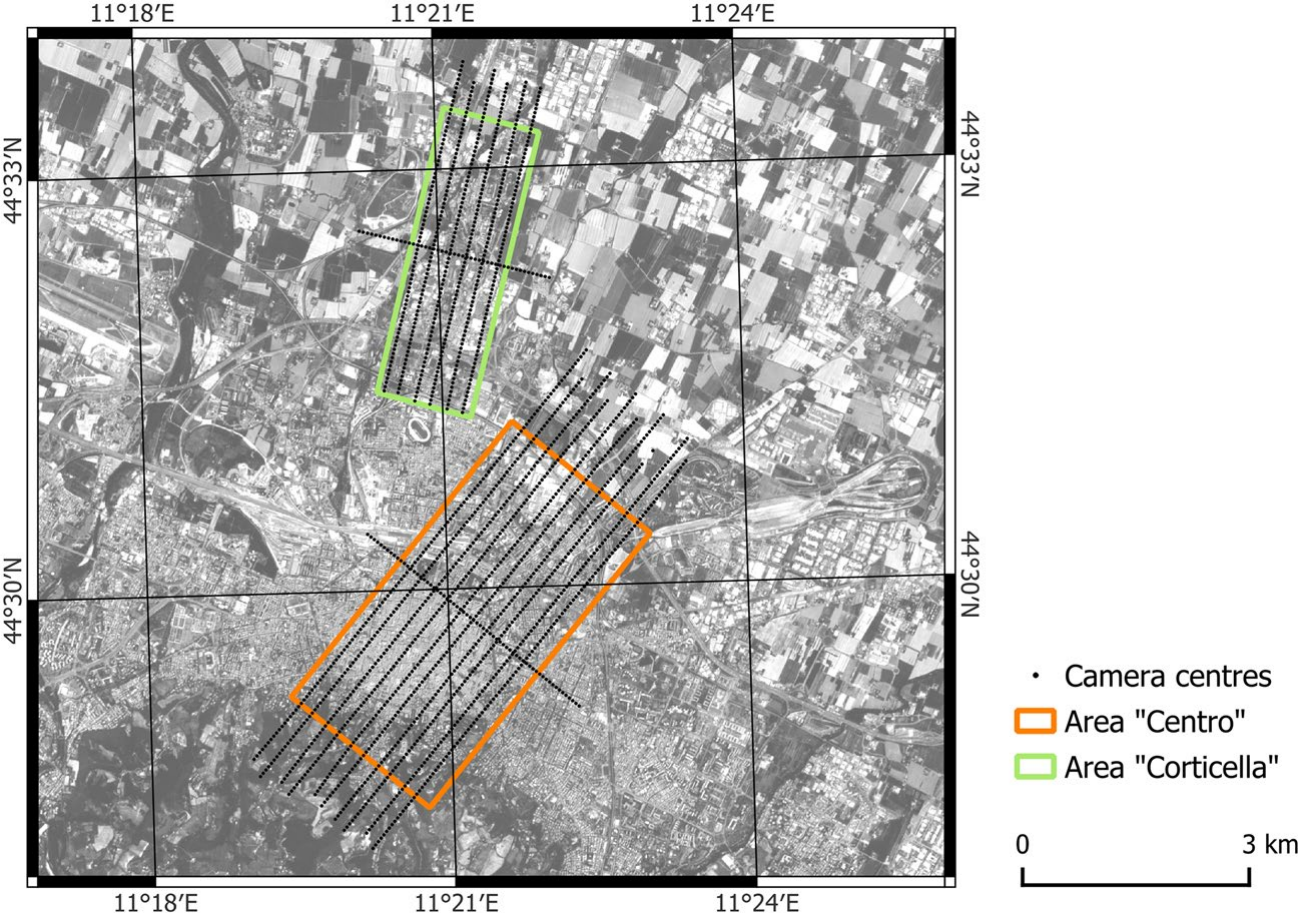
The two study areas in Bologna and the track of the aerial flights (map generated by QGIS software version 3.34^29^).

The proposed methodology is based on the datasets described in the following paragraphs and collected over the urban area of Bologna (Italy). More precisely, two areas of about 10 km^2^ and 5 km^2^ respectively were considered (Fig. 1). The first (called “Centro” in the following) covers the city centre and a business district, conversely the second is on a peripheral area (called “Corticella” district). The choice is made to encompass several kinds of urban textures and building types.

Two aerial flights were performed between 8 and 9 March 2017 to acquire two sets of thermal images, one diurnal, the other nocturnal. The first flight started at 12:15 p.m. (local time), while the second at 01:15 a.m. The solar altitude was about $40.65^{\circ }$ up to the horizon during the diurnal flight^30^. The flights lasted about 2 h, due to the complex management of the air traffic over the nearby international airport. The adopted thermal camera is a NEC TS9260, operating in the 7.5–13 $\upmu$m infrared interval, with a resolution of 640x480 pixels and a noise equivalent temperature difference of $0.06\,^{\circ }$C. The camera was rigidly mounted on a “Piper Seneca II” aircraft, together with an inertial measuring unit (IMU) and a GNSS receiver. The flight height of 800 m was designed to obtain a Ground Sampling Distance (GSD) of 0.5 m, given the narrow field of view of the optics ($21.7 \times 16.4^{\circ }$). During the surveys the acquisition of single frames was triggered at regular time intervals along the strips and the synchronisation with the GNSS and IMU units provided the position of the camera centres. Overall, more than 2,500 frames were acquired for each flight. Due to a temporary failure of the system, part of a strip is missing in the Corticella area.

A Digital Surface Model (DSM) obtained from panchromatic satellite images was available, at a spatial resolution of 0.3 m, obtained with the photogrammetric processing described in Mandanici et al.^31^. In summary, three WorldView-3 (WV3) stereo-pairs were acquired on-demand (one on 14 September 2017 and two on 20 September 2017); they were processed in the OrthoEngine tool of the Geomatica suite (version 2018, by PCI Geomatics), which implements the semi-global matching technique^32^. The image orientation is based on the coefficients of the rational polynomial model provided by the supplier, and refined with a first-order polynomial, whose coefficients were estimated using five control points and 73 tie-points. The generated DSM was validated with check points and other existing models of the area. The average error in elevation on roof surfaces and open spaces is 0.6 m, although severe discrepancies are reported inside urban canyons^31^.

Also, a map of the roofing materials over the entire city of Bologna is used, which results from the work by Trevisiol et al.^33^. In synthesis, an object-oriented supervised classification was applied on a WV3 acquisition of 20 September 2017, including both the eight multispectral and the eight SWIR bands, with the aid of the previously described DSM. The process was implemented in eCognition software (version 10.1, by Trimble). The map discriminates six macro-classes of widely used roofing materials, clay tiles, sheath, metal sheets, gravel, gravel tiles and other materials. The training is based on field inspections and drone surveys and the achieved overall accuracy is 91%. The observed errors are mainly due to artifacts in the DSM, or to particular coatings that can hide the distinctive spectral features of the roofing materials^33^.

**Figure 2:**
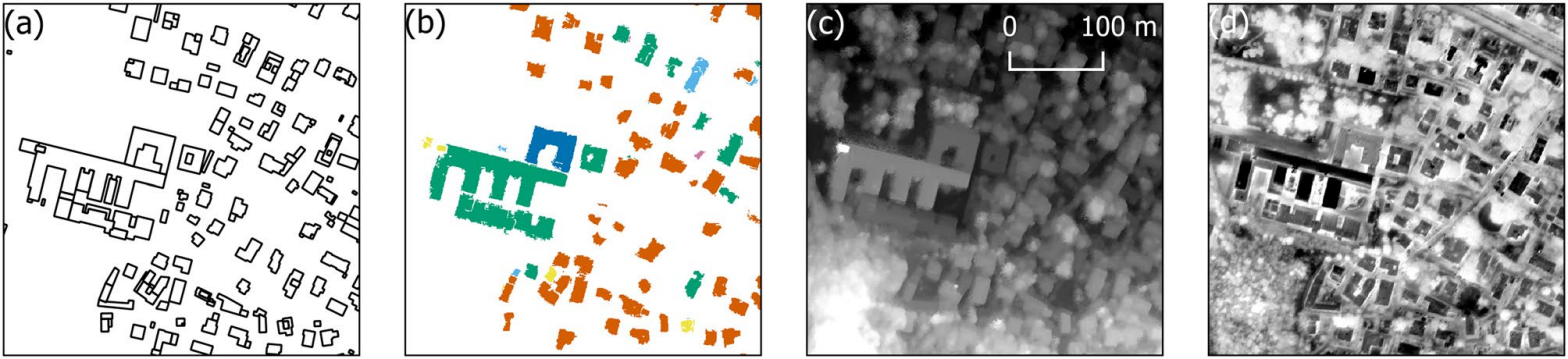
A small portion of the building layer of the technical cartography (**a**), map of the most common roofing materials (**b**), DSM (**c**) and uncalibrated thermal image (**d**) in the Centro area. (Maps generated by QGIS software version 3.34^29^).

Finally, the technical cartography of the municipality, which comes with a nominal scale of 1:2000 and a tolerance for planimetric coordinates of 0.5 m, was used as the base map for the building polygons.

All the data (Fig. 2) were coherently framed in the official geodetic framework for Italy, which is the ETRF2000 (epoch 2008).

## Methods

### Generation of the thermal mosaics

Thermal images generally show poor contrast because the temperature values of most of the objects vary in a narrow range. So, the entire dataset of diurnal and nocturnal thermal imagery was pre-processed.

A linear stretching has been used which maps temperature values as 16 bit integers, saturating the 2% tails of the overall histogram of the temperature values among all the images^34^. Coefficients of the linear transformation are stored in order to retrieve the original temperature values after the mosaicking phase. This stretching enhances the identification of corresponding features in overlapping images.

**Figure 3:**
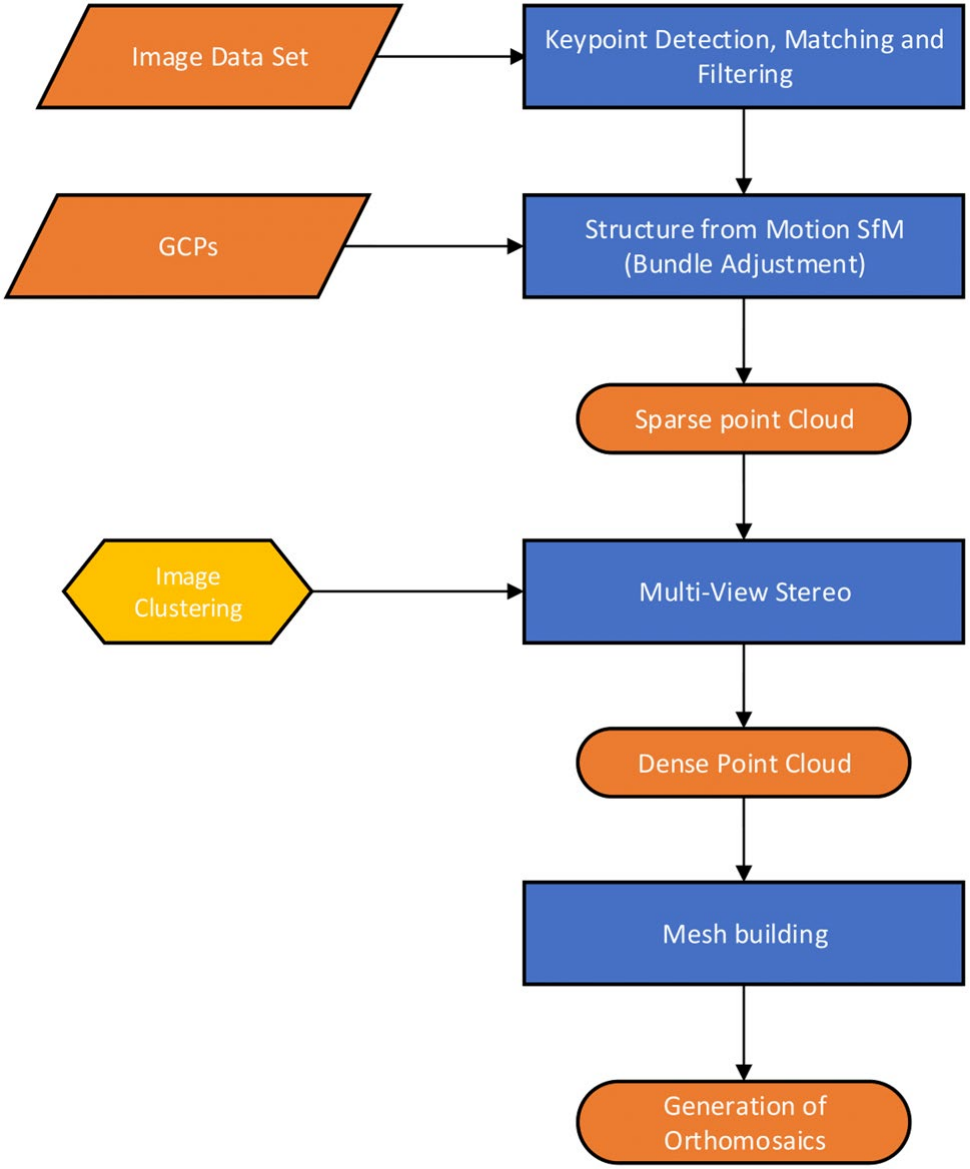
Workflow of the photogrammetric process by SfM.

Orthomosaics of the day and night flights were obtained by photogrammetry by adopting a Structure-from-Motion (SfM) approach^35^ based on the Bundle Adjustment (BA) algorithm. The workflow of SfM is reported in Fig. 3 and readers are referred to seminal publications in this field for more details^36–38^. In the bundle adjustment, ground control points are used to refine the camera positions and attitude at the time of the shot and to georeference the reconstructed three-dimensional model. In order to strengthen the estimation of the unknown parameters, especially in case of deficient acquisition geometries, additional constraints can be added in the form of tie points: these points are chosen by the user and collimated in two or more images in a completely manual or semi-automatic manner. The use of this computational method allowed to perform the self field calibration of the thermal camera adopted, introducing the geometric parameters of internal orientation of the camera as additional unknowns of the least-squares estimation^39^. The estimates obtained on the camera parameters from the day and night sets in Corticella area are listed in supplementary materials.

In the case of Corticella area 603 images acquired in the night flight and 562 acquired in the day flight were processed. The latter, as mentioned above, show uneven coverage due to a missing strip. For the Centro area, the photogrammetric process is described in Conte et al.^34^.

**Figure 4:**
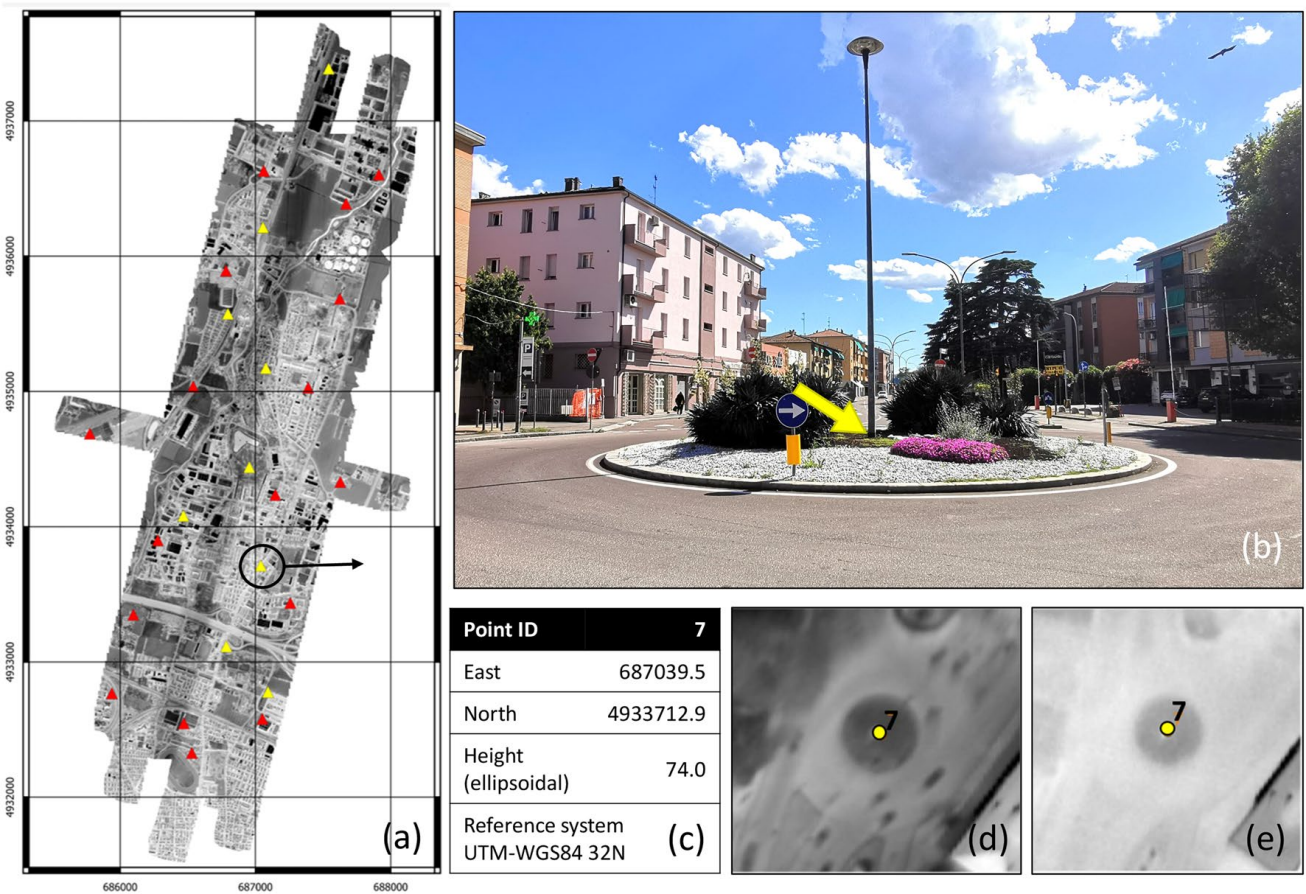
Constraints for the bundle adjustment: (**a**) the orthomosaic obtained from the night flight images with the GCPs (yellow triangles) and manual tie points (red triangles); the GCP 7 in a field picture (**b**), its measured coordinates (**c**), the same point in the diurnal thermal image (**d**) and in the nocturnal thermal image (**e**). (Maps generated by QGIS software version 3.34^29^).

Given the importance of achieving an optimal overlap between the final orthomosaics for subsequent analysis, special care was taken in the phases of image alignment and georeferencing. In the case of Corticella area (Fig. 4), 11 GCPs were surveyed using GNSS NRTK technique. A careful preliminary analysis of the thermal images allowed for the not easy identification of these points, which had to be well distributed in the area of interest, measurable in both day and night images, and also measurable in the fields with topographic instruments. In addition to the GCPs required for georeferencing, image alignment was optimized by manually inserting about 20 other markers (tie points) to create a homogeneous distribution of constraints and to strengthen the solution where the image coverage is more uneven, especially in the case of daytime flight.

The photogrammetric process—which was performed with Metashape software (version 1.8.4 Pro, by Agisoft LLC)—estimated good overall accuracy values for both sets: the reprojection error is significantly lower than one pixel both for GCPs and tie points (see supplementary materials).

Due to the difficulty of identifying a number of accurate control points on thermal images which are also measurable in the field, it was chosen not to use photogrammetric check points to validate the metric quality of the results. Indeed, for this study, the quality of the absolute georeferencing of the two orthomosaics is not as important as their co-registration (i.e. the absence of displacements or deformations between the two). Therefore, to check the quality of the photogrammetric processing, the differences between the positions of 29 points measured on both orthomosaics (hereafter called check points, even though they are not surveyed in the field) were investigated. This evaluation showed differences greater than the GSD (50 cm) and therefore not acceptable for subsequent analyses. This issue was solved in GIS environment through a finer co-registration, assuming the nocturnal image as the base and applying to the diurnal one a thin plate spline transformation. In the case of Corticella area, this transformation is based on a set of 54 points collimated in both the mosaics. After the transformation the residuals of the 29 check points are lower than 50 cm and the root mean square error (RMSE) equals 18.4 cm. As expected, the largest errors are observed near the north-east corner of the mosaic, where the geometry of the diurnal image block is weaker due to a missing strip.

The same co-registration refinement needed to be done on the Centro area. In this case, a total of 60 points were considered for the geometric transformation of the diurnal map. The goodness of the results was tested collimating other 28 points that showed an RMSE on the residuals of 21 cm.

### Temperature calibration

The temperature measured by the camera sensor does not coincides with the real temperature of the surfaces at ground level. The procedure to retrieve the actual Land Surface Temperature (LST) of roofs for both day and night was conducted by the following steps:

1. Generation of a Sky-View Factor (SVF) map for the study area. This was computed applying the model by Zacksek et al.^40^ and implemented in the Terrain Shading plugin for QGIS (version 0.9.3)^41^ on the digital surface model of the city, setting a search radius by 100 m.
2. Derivation of the atmospheric correction parameters. The empirical method presented by Mandanici et al.^42^ was used. In the study area a total of eight points were identified, corresponding to different types of materials, in which the temperature and emissivity values of the surfaces were measured through a thermal camera and a contact probe. A least-square adjustment was performed to find the optimal set of atmospheric parameters which minimize the residuals between the corrected temperature from the images and the measured values on those eight points.
3. Assignment of the emissivity coefficient to every roofing material that appears in the study area. The classification map obtained by Trevisiol et al.^33^ was used. The emissivity values are derived from direct measurements where possible or from literature data^42^, and are reported in Table 1.
4. Computation of the LST map for the day and night flights. Atmospheric parameters, emissivity and SVF maps were combined to compute the surface temperature for all the roofs in the study area according to the model described in Mandanici et al.^42^.

**Table 1:** Emissivity values for the considered classes of roofing materials.

Material class	Emissivity
Metal sheets	0.70
Clay tiles	0.88
Gravel tiles	0.90
Bituminous sheaths	0.91
Gravel	0.96
Other materials	0.87

### Temperature validation

**Figure 5:**
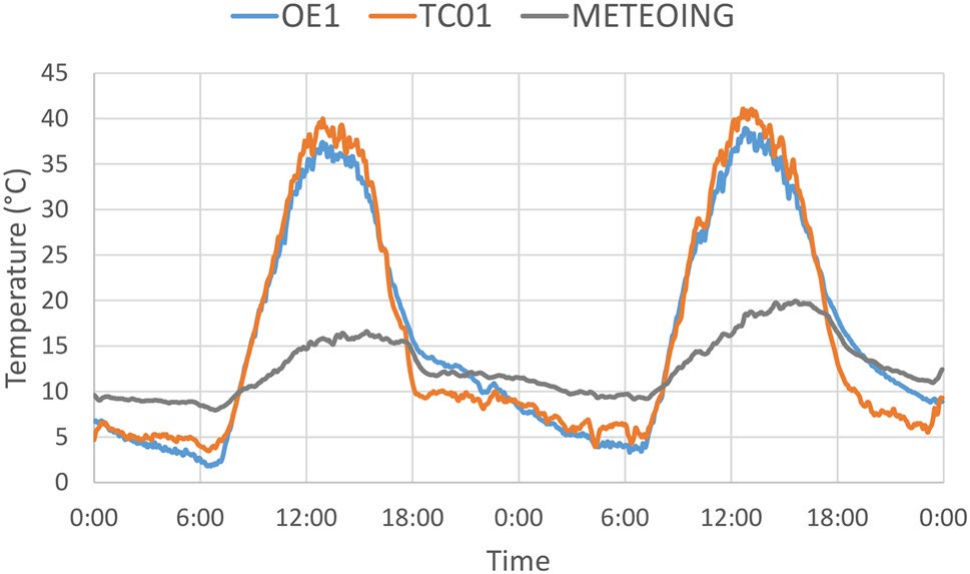
Thermocouple recordings on 8 and 9 March 2017 on the roof of the Engineering School in Bologna (OE1 on bituminous sheath and TC01 on metal sheet), compared with air temperatures measured by a local weather station (METEOING).

To check the accuracy of the temperatures obtained from thermal imagery, eight thermocouples (type K, with a tolerance of $1.1\,^{\circ }$C) were installed on accessible roofs with different materials. They were fixed on the surfaces with a transparent polyimide tape with silicone adhesive and connected to a datalogger that recorded the temperature every 5 min. A minimal quantity of thermal paste was added on rough surfaces. An example of logged temperatures is reported in Fig. 5.

### Albedo

The albedo map was obtained starting from the WV3 image of the case study area, according to Eq. (4). The image was acquired on 2017/09/20 at 10:25 UTC and the solar altitude was about $45.6^{\circ }$. The scene underwent a full atmospheric correction, which took into account the estimation of the solar irradiance at the time of acquisition. During this phase the solar elevation angle was taken into account, but no topographic correction was applied, leading to the computation of directional reflectance values. In this case the correction of the atmospheric effects was based on ground reflectance measurements, as described in Trevisiol et al.^33^. Also, the satellite image was acquired at 12:25 (local time), about 40 min before the solar noon, when the impact of solar elevation on albedo values should be minimized.

The surface spectral reflectance of every band was multiplied for a weight coefficient $w_\lambda$, which is calculated as the ratio between the spectral solar irradiance in each band and the sum of the irradiances of all bands^16^:

5 $$\begin{aligned} w_\lambda = \dfrac{I_\lambda }{\sum I_\lambda } \end{aligned}$$

The adopted values are reported in Table 2. Solar irradiance and WV3 bandpass functions are derived from the literature^43,44^.

**Table 2:** Solar irradiance values for each WV3 band expressed in W  m$^{-2}$  $\upmu$m$^{-1}$ and the weights adopted for the computation of the albedo, according to Eq. (5).

WV3 band	Solar irradiance	Weight
COASTAL	1757.89	0.1159
BLUE	2004.61	0.1322
GREEN	1830.18	0.1207
YELLOW	1712.07	0.1129
RED	1535.33	0.1013
REDEDGE	1348.08	0.0889
NIR1	1055.94	0.0696
NIR2	858.77	0.0566
SWIR1	479.02	0.0316
SWIR2	263.80	0.0174
SWIR3	225.28	0.0149
SWIR4	197.55	0.0130
SWIR5	90.42	0.0060
SWIR6	85.06	0.0056
SWIR7	76.95	0.0051
SWIR8	68.10	0.0045

### ATI computation

The ATI was computed on a pixel basis, using the Eq. (2), as well as the albedo values and temperature differences. In this case, the contribution of *S* in Eq. (2) is negligible: considering that the day of the flights was the 67th day of the year, Eq. (3) gives 0.997 for *S*. Therefore, the formula adopted here for the calculation of ATI was simplified, so it does not consider the seasonality.

Using the technical map of Bologna as the cartographic base, since it contains the location and geometry of the buildings in vector form, a mean value for the albedo and ATI was assigned to each building polygon through GIS overlay operations. Also, the predominant roofing material was assessed and the percentage of the area of the polygon covered by it. A threshold was set to exclude from the analyses those buildings showing a percentage of the dominant material lower than 80%. This way only homogeneous roofs were considered to compute the statistics on ATI representative for each class. In particular, the distribution of ATI values for each class are computed in both the study areas and the averages and standard deviations are computed after removing some outliers (the polygons where the standard deviation on ATI is higher than $10\,^{\circ }{\textrm{C}}^{-1}$ are removed).

## Results and discussion

### Day-night temperature variation

**Figure 6:**
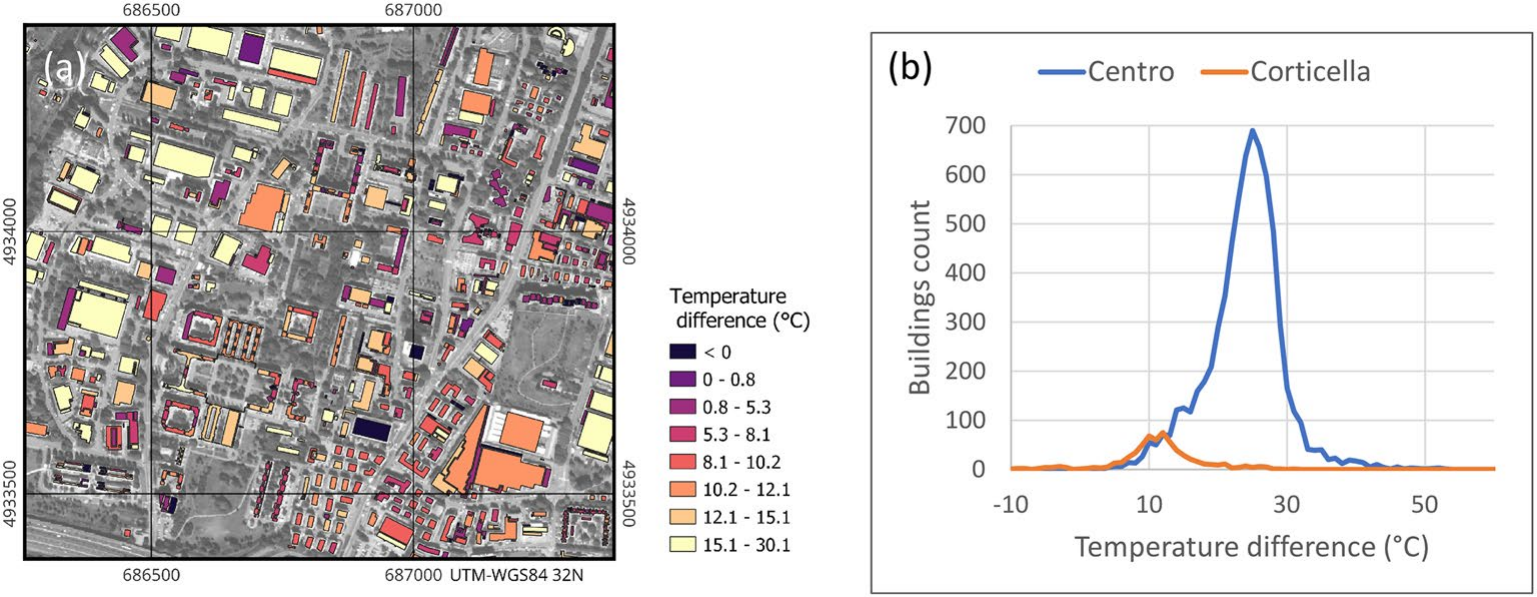
(**a**) Portion of the temperature differences map in the area of Corticella (map generated by QGIS software version 3.34^29^); (**b**) overall histograms of the computed values in the two study areas.

The proposed method allows the generation of a temperature difference map for all the roofs in the two investigated areas (Fig. 6). The distributions of the observed values have means of about 8 and $11\,^{\circ }$C and standard deviations of 7.3 and $5.7\,^{\circ }$C, for the areas of Corticella and Centro respectively, with a maximum of $30\,^{\circ }$C. A limited number of buildings (about 5%) show negative values, probably as a consequence of incorrect material classification (the accuracy of the classification is 91%^33^) or strong sky-radiance reflection.

The thermocouples installed on the roofs of some buildings allowed to evaluate the quality of the derived temperatures and temperature differences. Table 3 compares for each measurement site (identified by the point name, the area and roofing material) the values of day and night temperature extracted from the corrected images, with the corresponding values measured by the thermocouples at the time of the flights. The day-night temperature differences from both the data sources are also reported and their residuals are highlighted in the last column.

**Table 3:** Comparison between the temperature values obtained from images and measured by the eight thermocouples installed on some roofs. Residuals refer to the day-night differences.

Point	Area	Class	Thermal images			Thermocouples			
			Day	Night	Difference	Day	Night	Difference	Residual
OE1	Centro	Sheat	39.0	7.4	31.7	35.9	8.2	27.7	4.0
TC01	Centro	Metal	41.1	10.0	31.1	36.7	8.6	28.1	3.0
TC02	Centro	Sheat	36.4	8.1	28.3	33.0	9.1	23.9	4.4
TC03	Centro	Other	1.9	− 15.0	16.9	28.0	8.4	19.6	− 2.7
TC04	Corticella	Gravel tiles		7.2	–		6.6	–	–
TC06	Corticella	Gravel tiles		5.8	–		5.9	–	–
TC07	Centro	Gravel tiles	29.6	7.2	22.3	32.0	6.8	25.2	− 2.9
TC10	Centro	Sheat	43.9	5.9	38.0	47.0	7.5	39.5	− 1.5

The RMSE of the residuals in Table 3 equals $3.2\,^{\circ }$C and it is computed considering six locations and four material classes. Two further locations in the area of Corticella fall in the missing strip of the diurnal flight, so the check on temperatures is possible only for the night. The only anomaly is at point TC03, which is classified as “other material”; in this case the assigned emissivity is not appropriate resulting in a huge discrepancy between the computed and measured temperatures. However, this large discrepancy does not affect significantly the day-night difference, as proved by the residual which is of the same magnitude of the others. This is not surprising, because an error in the emissivity produces a systematic bias on the temperatures that can be in part mitigated when differencing. In addition, it is possible that the amount of longwave irradiance at the time of the two acquisitions (12 p.m. and 1 a.m.) is similar, as observed in previous works about sky radiance^45^. Indeed, under clear sky conditions, downward longwave irradiance is controlled by air temperature and absolute humidity^46^; the daily evolution typically shows a relative minimum in the early morning, followed by a growth until late afternoon, and finally values decrease in the last hours^47^. Overall, the observed residuals agree with the expected accuracies of the radiometric correction methodology, also considering the error propagation on a difference. Indeed, previous studies about LST determination in urban areas from aerial surveys found RMSE of $2.6\,^{\circ }$C^48^ and $3.3\,^{\circ }$C^49^.

### Albedo

**Figure 7:**
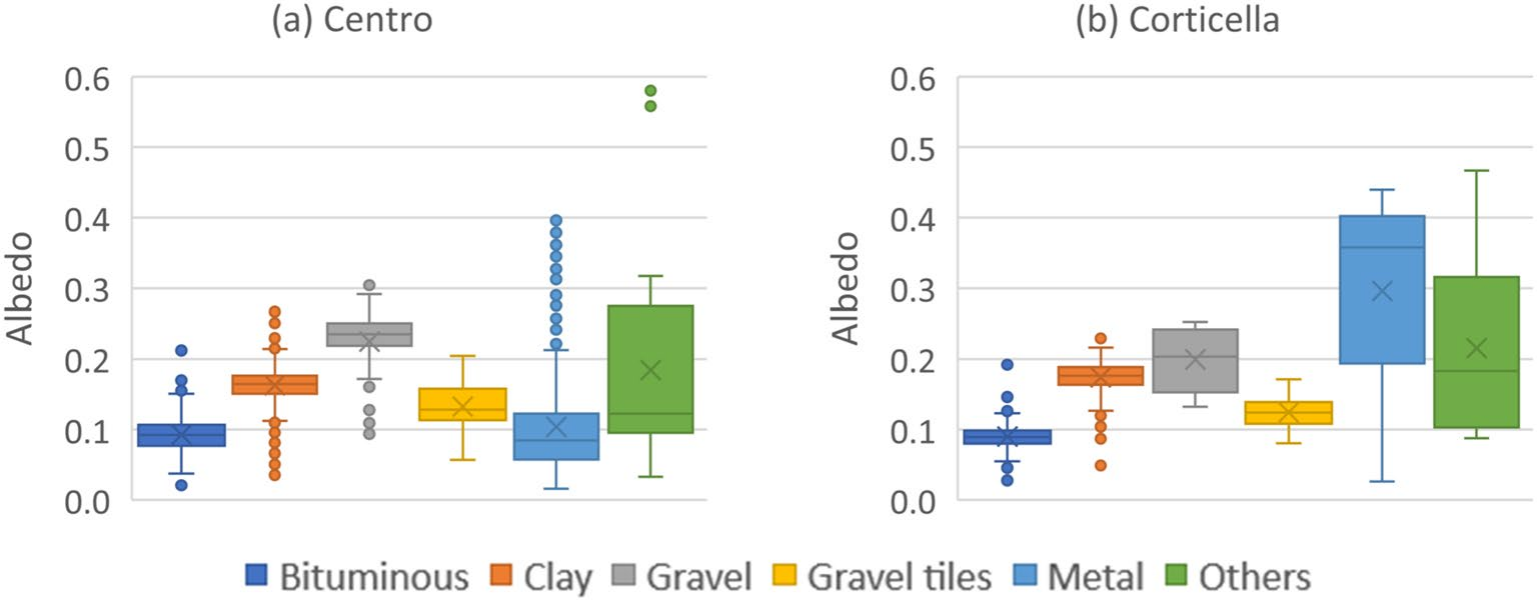
Statistics on the albedo for each class for the two investigated areas in Bologna. Boxes are delimited by the first and third quartiles, the horizontal line and the cross inside every box are the median and the mean respectively, the whiskers extend to the minimum and maximum elements that are within 1.5 times the interquartile range, and the dots are presumed outliers.

The albedo is computed for all the roofs in the two test areas and statistics on the obtained values are presented in Fig. 7. A strong coherence can be observed for clay tiles, bituminous sheath and gravel tiles; indeed, the variance is low and mean values for the two areas are practically the same. Conversely, metal and other materials classes show great dispersion of values and appear poorly replicable. For the other materials class the reason is obviously the small number of roofs and the large variability of roofing types. For the class of metal roofs, instead, the reason is likely to be related with the different paintings/coatings applied on the metal sheets, which are able to significantly change the overall albedo.

Furthermore, it is to be considered that albedo values measured in urban context show a dependence on solar zenith angle, and therefore on solar altitude^50^. However, albedo for urban surfaces is fairly constant between 20 and $65^{\circ }$ sun elevation^51,52^, as it is the case in the present work. Therefore, no compensation for this directional effect is performed here.

Probably the metal roofs class is too broad for this kind of applications and a finer discrimination in subclasses with different coatings is necessary. However, producing such a finer classification from remote sensing data over wide areas and without losing accuracy is still challenging. Furthermore, the comparison of the classification map used for the analysis with other classification techniques was not the aim of the present paper, but a comparison with new methodologies (such as deep learning) could be of interest for future studies. One option may be the use of hyperspectral imagery, but available satellite platforms does not support the required spatial resolution, therefore dedicated aerial surveys are necessary.

### Apparent thermal inertia

**Figure 8:**
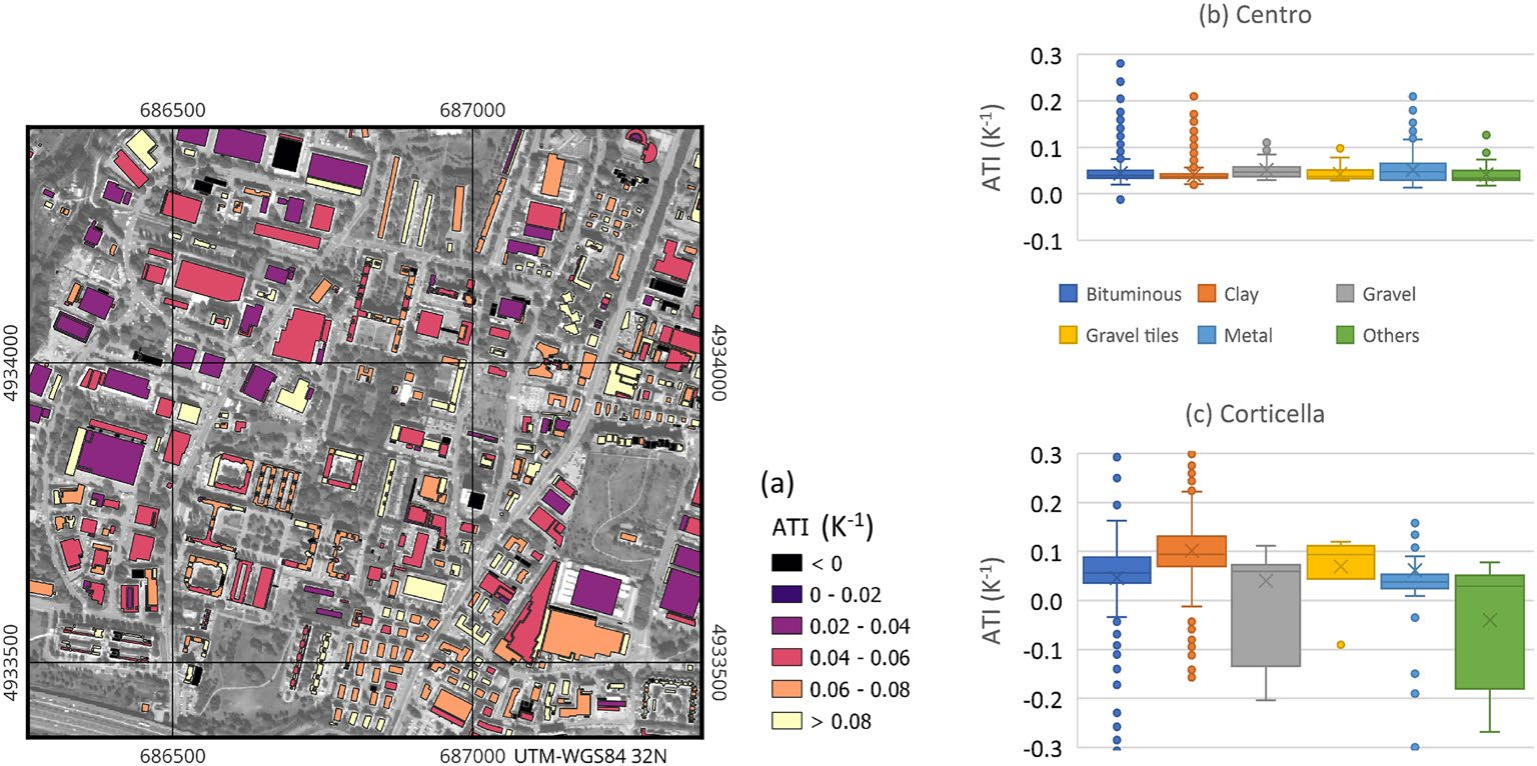
(**a**) Portion of the ATI map on the Corticella area (map generated by QGIS software version 3.34^29^); (**b**,**c**) statistics on the ATI for each class for the two investigated areas (box and whiskers plots as described in Fig. 7).

As described in the methods, a map of apparent thermal inertia of the roof surfaces is computed over the study area (Fig. 8a). Since at the time of the writing in the literature ATI values in urban areas are not reported with such a detail, it is impossible to compare the following results with previous analyses.

The analysis of the obtained results is possible only in a statistically aggregated form. Comparing the two study areas (Fig. 8b,c), the two corresponding average values for each class differ for a quantity that is lower than the standard deviation, demonstrating the robustness of the methodology. However, some discrepancies observed in the statistics deserve some discussion. In the Centro area, median ATI values are mostly the same for all the classes, in the range between 0.03 and 0.05 K$^{-1}$, while IQRs (Interquartile Ranges) are about 0.02 K$^{-1}$, except for metal roofs (0.04 K$^{-1}$). In the Corticella area, the observed values show higher variability, considering both median (from 0.03 to 0.09 K$^{-1}$) and IQR (from 0.03 to 0.14 K$^{-1}$).

The most pronounced discrepancy can be observed in the “Others” class. This can be explained by the more limited number of samples (six buildings only in Corticella and 28 in Centro), the heterogeneity of the materials included in this class and the limited accuracy of the emissivity value assigned to this class in the calibration process. This fact causes also negative values, which have no physical meaning, as a consequence of strong underestimation of the daytime temperature compared to nocturnal one. This fact can be caused also by a strong reflection of sky-radiance, especially for materials with low emissivity.

An exhaustive justification of the higher homogeneity of the observed ATI values in Centro compared to Corticella would require further investigations. At present, the following hypothesis can be formulated. In Centro, the urban texture and building typology is more uniform. According to a census carried out by the Municipality in 2011^53^, in the city centre 70% of the buildings were built in XIX and previous centuries; conversely, most of the units in Corticella were built in the second half of the XX century (60%), adopting a wider range of building typology and construction techniques. Also in terms of building use, the Centro is more uniform with 85% of residential units, while in Corticella they decrease to about 60%^53^.

A factor possibly affecting the intra-class variance is the slope of roofs. In fact, one limitation of the proposed methodology is that the contributions of slope and aspect are not compensated in the ATI computation (nor in the albedo). Different sun angles cause differential heating of the surfaces during the day and the same building may have two or more pitches with different exposures. In the city centre sloping roofs are more common than in Corticella area. In the literature about ATI, the topographic effects were considered only in some works about the mapping of Mars; in particular slope was found to affect the locally inferred albedo and temperature^54^. A methodology was developed on purpose to account for steep angles in the evaluation of ATI^24^. However, modelling slope effects on ATI in urban areas requires further investigations, due to their complex morphology, and this may be a future development of the proposed methodology.

A further reason for the observed intra-class variance may be the effects of ageing and deterioration of surfaces, which also affect the albedo^55^.

## Conclusions

The paper presented a methodology to map the Apparent Thermal Inertia (ATI) of roofing materials, exploiting the high-resolution thermal images obtained from two aerial flights over the city of Bologna. The two flights allow to estimate the temperature difference between day and night. The ancillary information needed for the temperature calibration are derived from dedicated ground surveys contemporary to the flights, while a classification of the main roofing materials was obtained from multispectral satellite data.

Structure from motion proved to be an effective tool for processing thermal images and the metric accuracy obtained is adequate for the purposes of this paper, especially for the night scenarios. However, to refine the co-registration between day and night temperature maps, diurnal orthomosaics were subjected to a coordinate transformation in GIS environment.

GIS confirmed to be a reliable tool for the integration of different geospatial data, allowing the calculation of key parameters for the analysis and to infer statistics of the results at single roof scale.

The obtained results in mapping albedo and temperature differences between day and night allowed the computation of the average ATI for each major class of roofing materials at building scale. In terms of ATI values, a relevant intra-class variability was observed, probably due to several factors, such as ageing effects, different building typologies or varying aspect of the sloping roofs. This variability—which cannot be explained only with differences in the material—makes the detailed mapping of ATI useful for studies on the urban micro-climate and on the urban heat island patterns inside urban areas.

Future studies may be focused on refining the presented methodology for the calculation of ATI and albedo, by taking into account roofs slope and aspect derived from higher resolution digital surface models. Further researches, which requires dedicated ground surveys, are also necessary to fully validate the estimated values of albedo and to check the correlation between the remotely observed ATI and the actual thermal inertia of materials.

## Data Availability

The datasets generated during and/or analysed during the current study are available from the corresponding author on reasonable request.
